# Recognition of Cosmic Ray Images Obtained from CMOS Sensors Used in Mobile Phones by Approximation of Uncertain Class Assignment with Deep Convolutional Neural Network

**DOI:** 10.3390/s21061963

**Published:** 2021-03-11

**Authors:** Tomasz Hachaj, Łukasz Bibrzycki, Marcin Piekarczyk

**Affiliations:** 1 Department of Signal Processing and Pattern Recognition, Institute of Computer Science, Pedagogical University of Krakow, 2 Podchorazych Ave, 30-084 Krakow, Poland; marcin.piekarczyk@up.krakow.pl; 2 Department of Computer Physics and Quantum Informatics, Institute of Computer Science, Pedagogical University of Krakow, 2 Podchorazych Ave, 30-084 Krakow, Poland; lukasz.bibrzycki@up.krakow.pl

**Keywords:** cosmic rays, CMOS sensors, mobile phones, citizen science, deep neural network approximation, transfer learning, image processing

## Abstract

In this paper, we describe the convolutional neural network (CNN)-based approach to the problems of categorization and artefact reduction of cosmic ray images obtained from CMOS sensors used in mobile phones. As artefacts, we understand all images that cannot be attributed to particles’ passage through sensor but rather result from the deficiencies of the registration procedure. The proposed deep neural network is composed of a pretrained CNN and neural-network-based approximator, which models the uncertainty of image class assignment. The network was trained using a transfer learning approach with a mean squared error loss function. We evaluated our approach on a data set containing 2350 images labelled by five judges. The most accurate results were obtained using the VGG16 CNN architecture; the recognition rate (RR) was 85.79% ± 2.24% with a mean squared error (MSE) of 0.03 ± 0.00. After applying the proposed threshold scheme to eliminate less probable class assignments, we obtained a RR of 96.95% ± 1.38% for a threshold of 0.9, which left about 62.60% ± 2.88% of the overall data. Importantly, the research and results presented in this paper are part of the pioneering field of the application of citizen science in the recognition of cosmic rays and, to the best of our knowledge, this analysis is performed on the largest freely available cosmic ray hit dataset.

## 1. Introduction

In this paper, we describe a convolutional neural network (CNN)-based approach to the problems of categorization and artefact reduction of cosmic ray images obtained from CMOS sensors used in mobile phones. As artefacts, we understand all images taht cannot be attributed to particles’ passage through the sensor but rather result from the deficiencies of the registration procedure. Our approach is based on the morphological properties of particle tracks rather than their physical interpretation, although some studies [1,2,3] associated certain shapes of tracks like spots, wiggles (which we here call worms), etc., with muons, electrons, etc. Unambiguous mapping between track shapes and radiation types, however, requires detailed studies of radiation propagation in a sensor of given geometry. Such studies are challenging for commodity devices and, to the best of our knowledge, had not yet been performed. Therefore, we take an alternative approach and categorize the registered events relying solely on their morphology. This study is timely and opportune as it is compatible with any future interpretation of a given track in terms of particle species.

**Figure 1:**
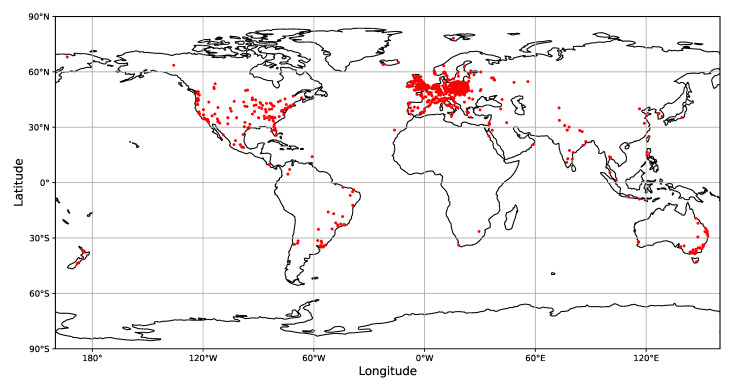
Locations of CREDO registered phone-based detectors.

In 1912, Victor Hess conducted a series of balloon experiments, revealing that the electric conductivity of the atmosphere increases with the elevation above the sea level. He boldly conjectured that the effect was due to the interaction of the atmosphere with the corpuscular charged particle radiation of extraterrestrial origin. More than 100 years after the discovery, due to their still undetermined origin, cosmic rays are being actively studied by astrophysicists. Further areas of interest include their implications for radiative safety [4], operation of electronic devices working both on Earth and in space [5,6], or even the earthquake prediction [7,8,9]. Of the several types of cosmic ray detectors [10,11], we focused on the semiconductor detectors [12]. Originally, they were conceived for measurements of particle energies, but with multi-sensor arrays equipped with many thousands of read-out channels and up to several hundred square meters of surface coverage, they became primarily used for particle tracking in nuclear and high-energy physics experiments [13]. Due to their low energy threshold, they also found applications beyond physical research, e.g., in medical imaging [14]. Notwithstanding their large number of applications, the basic physical processes upon which all semiconductor sensors are based are the same. Notably, the same physical processes are used in CMOS sensors applied in domestic electronic devices like video recorders or digital cameras used by mobile phones. The cameras of mobile phones are of particular interest for cosmic ray detection due to these devices’ ubiquity and network connectivity. Combining these features makes the mobile phones an ideal framework for creating the global network of radiation detectors coupled to central data storage. This idea underpinned several particle detection initiatives like CRAYFIS [15,16,17,18,19], DECO [1,3,20,21], and CREDO [22,23]. The analysis presented in this paper is based on the CREDO detection data set, as this is currently the largest publicly available data set of particle images obtained with mobile phones. The range of the CREDO worldwide device infrastructure is shown in Figure 1. Currently, the total number of registering devices is over 10,000 and is increasing.

### 1.1. State of the Art

As mentioned above, our goal was categorization and artefact rejection in cosmic ray images obtained from the CMOS sensors used in mobile phones by applying a two-dimensional analysis of the morphological properties to particle tracks. From the perspective of image processing and recognition, this problem should be solved by an algorithm from the group of algorithms devoted to the recognition of shapes and objects. Computer methods of shape feature extraction have been explored for many years. The most popular approaches are contour-based methods (i.e., Hausdorff distance, shape signature, boundary moments, spectral transform, shape invariants, etc.) and region-based methods (i.e., invariant moments, shape matrices, convex hull, etc.) [24,25,26]. In the last years, object recognition has evolved from early methods that used hand-crafted representations and descriptions to state-of-the-art deep-learning-based approaches. Especially, convolutional neural networks have become one of the most successful image-based pattern recognition methods [27,28,29,30]. A transfer learning approach is among most useful techniques for adapting pre-trained CNN architectures to other image domains [31,32,33,34]. With the aid of transfer learning, it is possible to train an effective deep neural network (DNN) architecture with a limited number of training samples because it is possible to reuse previously trained kernels. DNN can also be successfully used in approximation tasks using uncertain data [34,35,36]. In practice, in some cases, it is possible to use the previously trained convolutional layers of a neural network as the input of a deep learning architecture. By using those pretrained layers, time and resources can be saved because rather than training from scratch, already available knowledge can be used.

### 1.2. Study Motivation

Conventional cosmic ray detectors range in scales from several centimeters square to about 3000 km square, like in the case of the Pierre Auger observatory [10]. Even such vast facilities must be considered of limited coverage, so to increase the number of registered showers, either the detector’s surface should be increased or it should be run longer. Both options are economically prohibitive. So, the idea behind projects like CREDO is to trade the very limited coverage of a single phone sensor, which is of the order of a few millimeters square, for the huge number of particle-detecting devices scattered worldwide. This is an example of a citizen science project, where the research infrastructure is contributed by interested but not necessarily scholarly affiliated members.

However, the practical implementation of this attractive concept meets several difficulties that need to be properly considered. First, contrary to detectors working as parts of dedicated research infrastructures, the geometries, up and down times, and working conditions of individual sensors remain uncontrolled. Various devices’ responses to similar particle signals may vary considerably depending on sensor geometry (height, width, and depth), noise level, and particular noise reduction algorithms implemented in the device (for a detailed discussion of sensor working conditions, see [23]). To enhance the participants’ activity, the project relies on the gamification of measurements, with the adverse effect of the possibility of users cheating (i.e., deliberately producing artefacts). Thus, the scientific quality of a given device output generally needs to be evaluated by individual inspection, which is possible to only a limited extent, as currently there are over 18 million registered events and this number is expected to increase by two orders of magnitude [23]. The search for anomalies requires a flexible and adaptive approach.

Therefore, methods have to be developed for automatic artefact rejection as well as searching for particular signals of interest. In this context, the machine learning methods and convolutional neural networks are particularly suitable. Importantly, the research and results presented in this paper are in the pioneering field of the application of citizen science in the recognition of cosmic rays and, to the best of our knowledge, this analysis is performed on the largest freely available cosmic ray hit dataset.

From the perspective of motivation, the methods and specific tested architectures in our work are similar to those of [1] (project DECO). However, there are significant differences in image labeling for the classification purpose between our data set and that from DECO, which has convinced us that it is worth trying a different approach than the one proposed so far. According to [1], the class was also assigned by eye, by multiple people, and if humans disagreed on the classification, which occurred 10% of the time, the image was labeled as ambiguous and excluded from the training set. In our case, as can be seen in Table 1, about 66% of images were labeled unanimously by all judges. There might be two reasons for that: either the DECO data set is higher quality than ours or, more probably, a different labeling approach was undertaken; for example, in our case, judges did not contact each other. How many judges participated in labeling the DECO data set was not specified. The large ambiguity in the data set is, in our opinion, cannot be ignored. Moreover, we can take advantage of it. Remember that uncertainties provide additional information about inter-class similarity.

## 2. Materials and Methods

### 2.1. Problem Formulation

As mentioned above, it is currently not possible to associate unambiguously particular particle types with track morphologies. Therefore, we proceedws in a general way and defined 3 morphological categories, which we dub spots, tracks, and worms, the latter being tracks with one or more wiggles of sufficiently large curvature for them to be visually distinguishable from tracks. The common feature of these 3 categories of signals is that they are quasi zero-dimensional (point-like) or one-dimensional (line-like). This is in line with the physical intuition that the microscopic objects colliding with the sensor’s surface are able to deposit the charge within a small vicinity of the collision point. This entails point-like events if the particle hits the sensor at the angle close to 90${}^{\circ }$ and line-like events if the particle hits the sensor at smaller angles. Additionally, we defined the artefact category that encompasses all events not satisfying the above requirements, i.e., those featuring large widths (being effectively two-dimensional) or related to too-large energy/charge deposit in the sensor. The approach that was undertaken to overcome the ambiguity of assigning images to a certain class was to ask a group of judges to assign each image to one of the four classes. Each judge could assign an image to only one class. They could also skip voting for certain images if unsure as to which class it should be assigned. According to this, if there are *n* judges, no more than *n* votes could be cast to a single class. It is also possible that a certain image would have zero votes cast on all classes. This situation occurs when all judges decide to skip voting this image when they are uncertain as to what class it belongs. We discuss the data set that was used in this experiment in Section 2.3. In summary, a labelled data set contains pairs: an RGB image *I* and a 4-dimensional vector of votes $\bar{v}$, each coordinate of which is the number of votes cast to a certain class.

The problem we aimed to solve was assigning a certain shape that is registered by the detector to one of the four classes: spots, tracks, worms, or artefacts. This is a classification problem, but we did not have ground truth image data labels defined as a crisp set. Due to the subjectivity of judges’ decisions, it is possible that each image was assigned to more than one class. We could have filtered out all ambiguous data and leave only images that were unequivocally assigned to a single class; however this binary approach would have caused the loss of some important information about visual class similarities. Due to this, to model the uncertainty in judges’ voting, we formulated this problem as an approximation rather than classification. Let *I* be an input image in the RGB color scale. To each image *I*, we want to assign a 4-dimensional real-valued vector with non-negative coordinates $\bar{p}$, which approximates the potential voting of judges, using a certain approximation function Φ. Each dimension of the vector represents the number of votes that judges cast for a certain class.

(1) $$\Phi \left(I\right)=\bar{p}$$

To make the approximation independent of the number of judges that participated in data set preparation, we also assumed that coordinates of vector $\bar{p}$ are scaled in range [0,1], where 0 means that no judge voted for a certain class, while 1 indicates that all judges voted for it. We can easily transfer the votes of the judges from vector *v* to *p* by division of each coordinate of *v* by the number of judges *k*.

(2) $$\bar{p}=\frac{\bar{v}}{k}$$

Vector $\bar{p}$ is neither normalized nor do its coordinates sum to 1 intentionally. Finally, we have the following data set *D*:

(3) $$D=\{\left(I_{i},\bar{p_{i}}\right);i=\left[0,\cdots ,n\right]\},$$

where $I_{i}$ and $\bar{p_{i}}$ are the *i*th image and the judges’ labelling of the image, respectively; *n* is number of images in the data set.

### 2.2. Approximation of Uncertain Class Assignment with Deep Convolutional Neural Network

The data set in the form presented in Equation (3) can be easily adapted to a machine learning framework. As indicated in Section 1.1, the state-of-the art approach for image embedding is the application of convolutional neural networks. We can either design a dedicated architecture that, after training, will generate valuable feature vectors, or use a pretrained model and retrain its non-convolutional layers using transfer learning. The first option requires a relatively large data set of example images, which might be difficult to manually label by judges. Because of this, we decided to use the second approach and adapt already trained network models. The second approach has a very important advantage: a pretrained convolutional network has many specialized filters that, in many cases, can be adapted to detect sophisticated objects (and shapes) in input images. The output of each CNN was processed by a global average pooling 2D layer and then propagated to the next layers. Because, as already mentioned in Section 2.1, we wanted to model an approximation rather than perform classification, we followed convolutional DNN in two layers: a dense (fully connected) layer with 128 neurons with ReLu activation function and the final dense layer with four neurons with a sigmoid activation function. A ReLU activation function is defined as [37]:

(4) $$relu\left(x\right)=\left\{\begin{array}{ccc} x & if & x>0\\ 0 & if & x⩽0 \end{array}\right.$$

**Figure 2:**
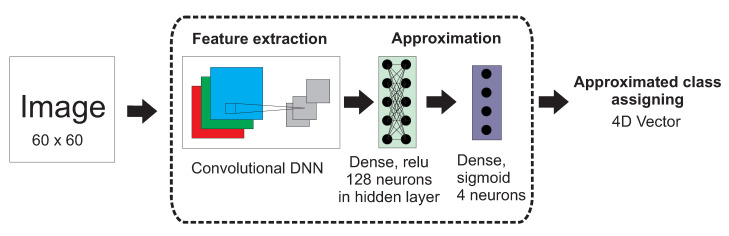
The architecture of the proposed deep convolutional neural network for uncertain class assigning.

A sigmoid neurons layer provides the opportunity for signal approximation. The schematic diagram of the system architecture is presented in Figure 2. The input dimension of the image was set to 60 × 60 (see Section 2.3).

The proposed approximator was trained using a first-order gradient-based Adam optimizer [38] with a mean squared error loss function; CNN layers weights remained fixed.

(5) $$MSE=\frac{1}{n}\sum_{0}^{n}{\left(\bar{p_{i}}-\bar{c_{i}}\right)}^{2},$$

where $\bar{c_{i}}$ is the prediction returned by the network.

Several CNN-based feature extractors were considered, namely Xception [39], DenseNet201 [40], VGG16 [41], NASNetLarge [42], and MobileNetV2 [43]. Each network was pretrained on the ImageNet data set [44]. We chose a well-established and verified CNN model pretrained on various complex objects that are present in the ImageNet data set. The CNN architectures seem to be excessive for potentially fairly simple, highly processed images; however, the images were gathered by a large network of CMOS sensors that have nonuniform hardware and software parameters and they were not primary designed as cosmic rays detectors. As such, although our data set contains 2350 images assigned to four classes by the judges, they are highly diverse, which is reflected by the ambiguous assessments of judges. As such, we decided to use embedding generated by general purpose pretrained CNN models that have convolutional multi-scale filters capable of modeling various possible typologies that might be registered by CMOS detectors. Our data set might not be large enough to train CNN-based embedding layers from scratch.

The cascade of convolutional filters with an architecture based on VGG16 was also used previously [1] and the authors decided to train it from scratch. As such, Winters et al. [1] had to undertake extensive data augmentation, which was not required in our case, because we adapted the VGG16 weights using transfer learning. As opposed to Winter et al. [1], we also applied basic image processing, which excluded salt-like noise from the input images.

The next problem that had to be addressed was assigning the class based on the certain result of voting $\bar{p_{i}}$. The most straightforward approach is to assign an image to the class that is represented by a coordinate of $\bar{p_{i}}$, which has the maximal value. If more than one coordinate has the same value, an image is assigned to a random class from those top-voted. This approach, however, could lead to situations where some images, for which approximation represents highly uncertang of judges, will also be assigned to a class. For example, if there is the same distribution of votes to each class, the assignment will be random.

(6) $$C_{i}=\max_{id}\bar{p_{i}},$$

where $C_{i}\in \left\{Dots,Lines,Worms,Artefacts\right\}$.

For DNN-based approximation, it is hardly possible that two neurons generate an identical response; however, it is possible that a final layer will generate a vector with all coordinates being, for example, close to zero and simultaneously not much differing from each other. We intentionally did not apply a SoftMax activation in the last layer as in Winter et al. [1] because this approach is unsuitable for simulating (approximating) the voting of separate judges. A SoftMax activation function is defined as:

(7) $$softmax\left(x_{i}\right)=\frac{e^{x_{i}}}{\sum_{j}e^{x_{j}}},$$

where SoftMax is the exponent of the input $x_{i}$ divided by a sum of the exponents of inputs $x_{j}$ [37].

Instead of applying SoftMax, we preferred to use a threshold scheme with a border (threshold) parameter *t*. In this scheme, the image $I_{i}$ is assigned to the class if and only if a maximal value of vector $\bar{p_{i}}$ coordinate is greater than *t*:

(8) $$C_{i}^{t}=\left\{\begin{array}{ccc} id & if & \max_{id}\bar{p_{i}}>t\\ \emptyset & if & \max_{id}\bar{p_{i}}⩽t \end{array}\right.,$$

where ∅ means that the classifier left the object without assigning it to any class.

### 2.3. Image Data Set

As of October 2020, there were about 18 million events registered in the CREDO database from 16,000 devices scattered around the world. Of them, about 5 million of events meet the requirements allowing to qualify them as visible, which, among others, means that complete event metadata are recorded in the database and the integrated brightness (related to the energy deposit) falls below the fixed threshold [23]. Of the visible events, we selected the data set of 2350 60 × 60 RGB images for this research. These images were subject to classification by 5 judges. After applying the class assignment method, 527 images were assigned to the spot class, 360 to the track class, 305 to the worm class, and 1158 to the artefact class.

The data set preparation procedure consisted of the following steps:

1. Selection of the subset of the trustworthy devices operating in controlled conditions;
2. Taking the image sample from trustworthy devices containing all morphologies of interest;
3. Assigning the dataset elements to four classes with the help of 5 judges with the majority vote while retaining the number of votes cast for each class.

As there were potentially a few sources of artefacts like hardware malfunction, insufficiently tight lens covering, or outright user cheating, we decided to introduce the notion of trustworthy devices. These are devices that performed the experiment in controlled conditions. To create a representative dataset for this article, we used data from our own devices that were run and operated under the supervision of CREDO researchers. We used the signals only from those devices so that the possibility of using cheating-affected data was entirely eliminated. Table 1 presents the distribution of votes for the classes in the data set.

### 2.4. Image Preprocessing

**Figure 3:**
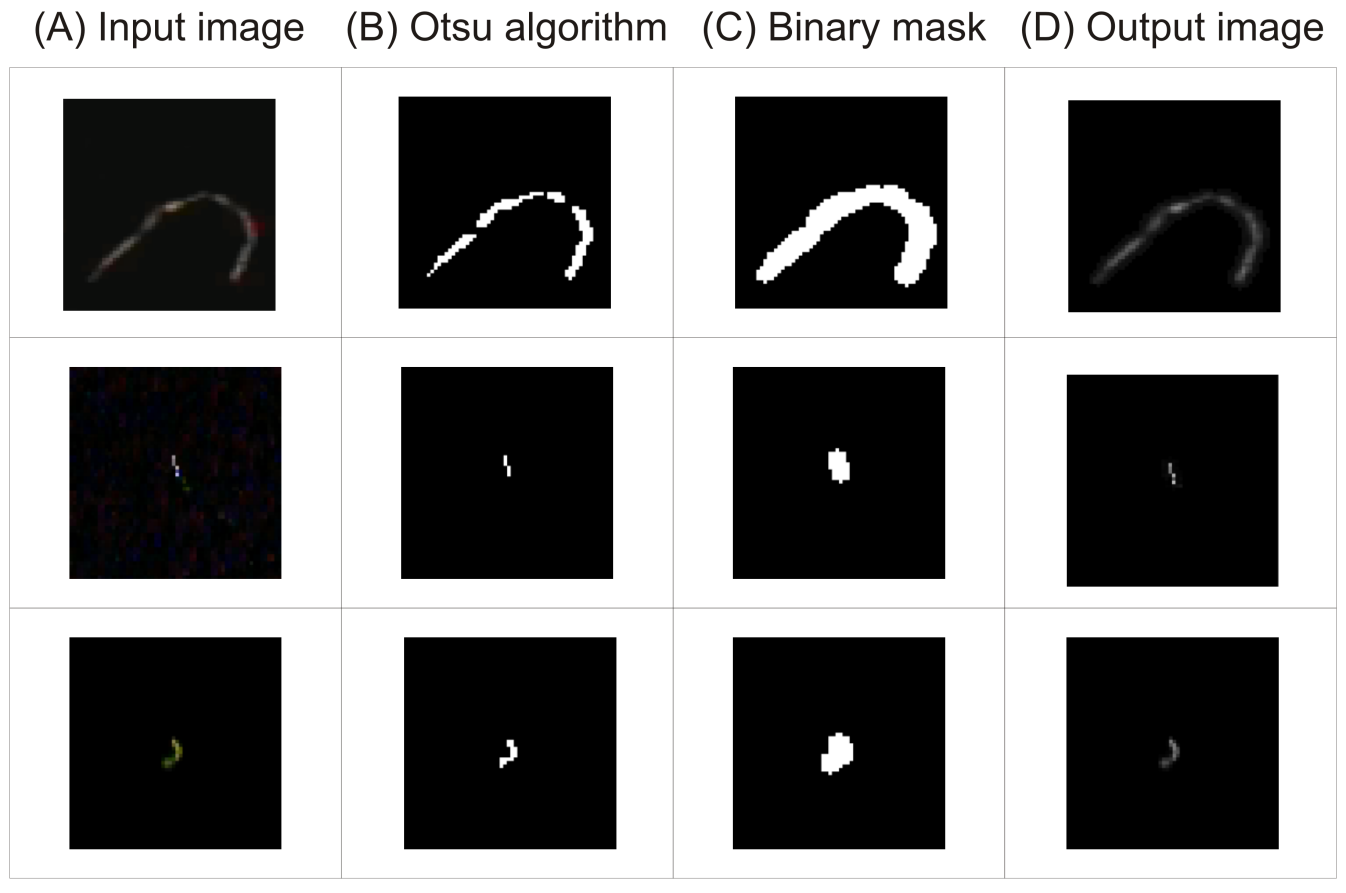
Image preprocessing pipeline. Each row represents a separate image. Each column showsthe final result of a single preprocessing transformation performed on the image. The followingprocessing steps are included: input image loading with conversion to grayscale format (**A**), automaticthresholding via the Otsu algorithm (**B**), creation of a binary mask (**C**), and definitive filteringoverlaying the binary mask on the grayscale image (**D**).

Before the image is processed by the CNN, some initial preprocessing is performed. The goal of preprocessing is to remove all objects but the signal of interest from the image set. The signal of interest is defined as white objects with sufficiently high color value in the RGB space. Preprocessing is performed with the following image processing steps (Figure 3):

1. Let *I* be an input image in the RGB color scale (Figure 3A). First, the image is converted to gray scale. The gray value is calculated as the linear combination of the weighted RGB channels values by a standard OpenCV 4.2.0.32 function (see details in source code).
  $$I_{g}=gray\left(I\right)$$
2. An object of interest is detected by maximizing the separability of the resultant classes in gray levels using an Otsu algorithm [45] (Figure 3B). The result is stored in binary mask.
  $$M_{ask}=Otsu\left(I_{g}\right)$$
3. The binary image $M_{ask}$ is dilated and then opened using image morphology operations [46] with an elliptical kernel with a diameter of 5 pixels. After this operation, the objects detected by the Otsu algorithm have slightly increased their borders and nearby objects are merged together. Opening also removes small holes in regions (Figure 3C).
  $$M_{ask1}=Dilate\left(M_{ask},kernel\right)$$
  $$M_{ask2}=Open\left(M_{ask1},kernel\right)$$
4. The final image $I_{p}$ is generated by extracting from the gray scale image $I_{g}$ only those pixels that are in the non-zero region of the binary mask $M_{ask2}$. The rest of the pixels in $I_{g}$ are set to zero (Figure 3D).
  $$I_{out}=I_{g}\&M_{ask2}$$

The above pipeline is repeated for each image $I_{k}$ from the data set described in Section 2.3. The set of output images $I_{out_{k}}$ is presented as an input image to the CNN. The role of the above image processing pipeline is to mime the procedure that is performed by each judge, who assigned images to a certain class. Judges only considered the curvatures of the object; the backgrounds were irrelevant to them. The proposed algorithm generates a binary mask whose role is to enhance only the object detected by the Otsu algorithm and the small surroundings of those objects, because the borders of those regions are blurred. We chose a kernel with very small diameter (5), which has the potential to fill holes with a diameter of about 3 pixels and to remove salt-like artefacts. Due to this small kernel diameter, the curvature of the detected objects remains the same. Perhaps it is possible to skip the above data processing; however, all background noises will be present in CNN embedding, which will disturb the final recognition process.

## 3. Results

The proposed image preprocessing and neural network approximation pipeline introduced in Section 2.2 and Section 2.4 were evaluated on the data set discussed in Section 2.3. The solution was implemented in Python 3.6. Among most important packages that were used were Tensorflow 2.1 for machine learning, deep neural networks Keras 2.3.1 library, and OpenCV-python 4.2.0.32 for image processing. Additional data evaluation was conducted in R version 3.6.2. The research was computed on a PC with an Intel i7-9700F 3.00 GHz CP, 64 GB RAM, NVIDIA GeForce RTX 2060 GPU, and operating on Windows 10 OS. Both source codes and data are available for download from an online repository (https://github.com/browarsoftware/credo_dnn_rays_recognition, accessed on 10 March 2021).

The training parameters were set to 4000 training epochs and batch size to 64. The learning rate for the first 2000 iterations was 0.001 and for the next 2000 was 0.0001. The learning rate governs the step size of the gradient descent method (see parameter α in [38]). The data set was split into a training data set that contained 90% of the objects (2115 images) and a validation data set with 10% of the objects (235 images). Each network with different CNN feature extractors was evaluated 10 times on different random data sets. Each training data set had 2115 elements randomly chosen from the 2350 images (without replacement); the remaining 235 images were assigned to the validation data set. In case of tied-voting by the judges in Equation (6), we did not re-randomize classes assigning for those ten sets. The results were averaged and the numbers in all tables are percentage values.

**Figure 4:**
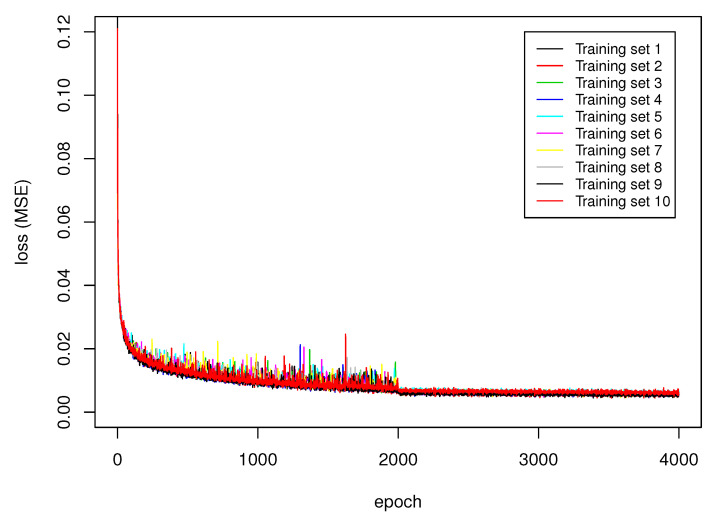
Changes in the loss (mean square error (MSE)) function during the training of the neural network with the VGG16 input layer. Time is measured in epochs. Each line represents a different random training set. After 2000 epochs, we observed smaller variance in the loss caused by reducing the learning rate from 0.001 to 0.0001.

Table 2 presents the recognition rate and mean square error of networks with various input convolutional architectures. The recognition rate is the total number of correctly identified images from the validation data set divided by the total number of images in the validation data set [47]. The highest recognition rate was obtained using VGG16. The second highest recognition rate for DenseNet201 differed only by 1.1% and had slightly smaller variance. Both networks have the smallest mean square error (MSE). During the training of all networks, the loss (MSE) function was minimized until reaching a certain value, which depends on the input CNN, the initial random weights choice, and the training data set (Figure 4). The relatively low variance of the values in Table 2 indicates, however, that the choice of CNN has the strongest impact on the overall results, and the network effectiveness is robust to initial random parameters and training data set choice.

**Figure 5:**
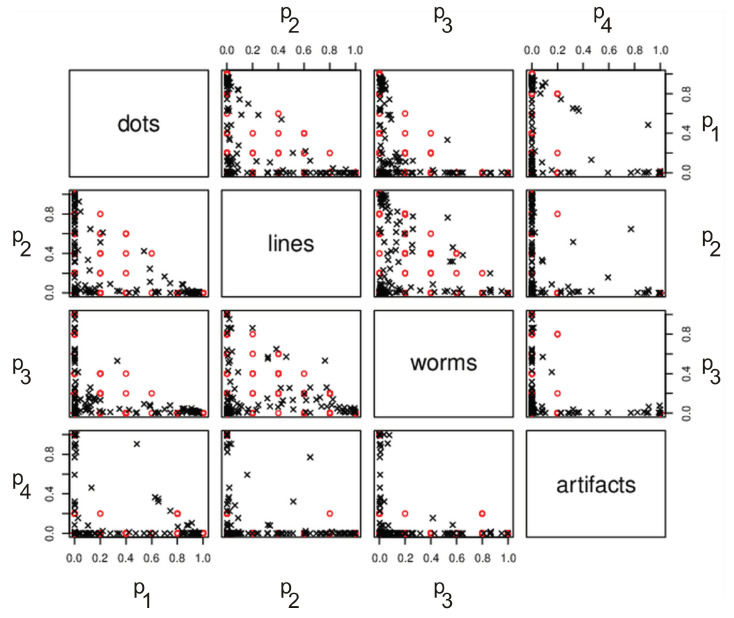
The pairs plot showing the bivariate relationships between all pairs of variables in one of the validation data sets. The pairs plot is represented as scatterplots between all pairs of these variables. In the first line, there is a scatter plot of spots and tracks, then one of spots and worms, and then one of spots and artefacts. The second row presents tracks and spots (symmetric to the first), tracks and worms, and so on. For a detailed description of the pairs plot, see [48]. Red dots are judge-labeled while black crosses are predicted values. Predictions were performed using a neural network with the VGG16 input layer. Values on the axis are the coordinates of vector $\bar{p}$ (see Equation (2)). For example, $p_{1}=1$ means that all judges voted for dot and $p_{2}=0.5$ means that half of the judges voted for line.

Figure 5 presents a pairs plot showing the bivariate relationships between all pairs of variables for one of the validation data sets. Red dots are judge-labeled while black crosses are predicted values. Predictions were performed using a neural network with the VGG16 input layer. As observed, judge-labeled values are obviously discreet; because of that, most values overlap and are represented by the same points in space.

Table 3, Table 4, Table 5, Table 6 and Table 7 present the confusion matrices of the networks with input convolutional architectures VGG16, NASNetLarge, MobileNetV2, Xception, and DenseNet201, respectively. Matrices are row-normalized and each row represents a judge label. Columns represent the predicted label. In all cases, over 93% of artefacts were correctly classified. The true positive rate of the rest of the classes depended on type of input convolutional neural network. The highest recognition rates for spots, tracks, and worms were obtained using VGG16. The highest recognition rate for artefacts was obtained using the DenseNet201 architecture; however, the difference between this network and VGG16 was only 0.68% with similar variance values.

**Figure 6:**
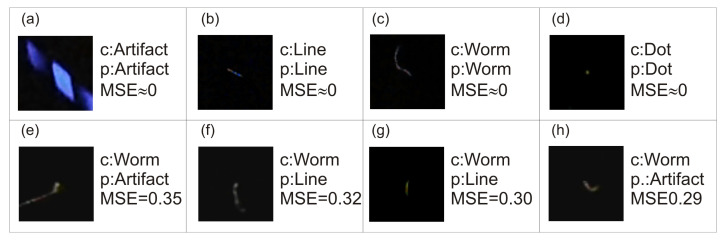
Example of best and worse approximations. C means correct (judge labeled) class, p ispredicted class, and MSE is a mean squared error between judge-labeled value and predicted value.Predictions were performed using a neural network with the VGG16 input layer. The MSE of theimages in the first row is below 0.005. The images (**a**–**d**) represent correct assignments, while (**e**–**h**) illustrate cases of misclassification.

Figure 6 visualizes an example of the best and worse approximations for predictions performed using the neural network with the VGG16 input layer.

Table 8, Table 9 and Table 10 present confusion matrices after applying the threshold scheme (8) with various thresholds to the network with the VGG16 features generator. Only VGG16 was evaluated because it proved to be the most reliable in previous experiments. The threshold scheme eliminates less certain predictions with a threshold of *t*. The table captions provide information about threshold *t*, validation data that remain after applying the threshold, data that remain after being split into classes, and overall recognition rate.

## 4. Discussion

As shown in Section 3, the proposed deep convolutional neural network architecture is capable of approximating uncertain class assignments that were performed manually by a group of judges. There are two measures we used to evaluate our solution: RR and MSE. Although there are a large number of trainable parameters in classification layers, the high recognition rate evaluated in 10-fold cross-validation assures that the network was not overtrained and still has generalization ability. All convolutional feature extractors have relatively small MSE, while VGG16 and DenseNet201 seem to be the best for the task. The value of MSE corresponds to the recognition rate of the network: the smaller the MSE, the better the recognition rate of the network. This is an important finding because it indicates that the uncertainty modelling of judges’ decisions was correctly designed (Table 2). The training of the proposed architecture is stable and follows expectations. The lowering of the learning rate value stabilizes the variation in the loss functions and slightly decreases the MSE (Figure 4). Lowering the learning rate after a certain number of iterations of the gradient-descent method lowers the influence of the gradient on the final solution. This allows for a better adjustment of the solution to the local minimum. According to confusion matrices presented in Table 3, Table 4, Table 5, Table 6 and Table 7, the artefact class was the easiest to recognize. This is probably because those images differ the most from other classes despite artefacts potentially having various forms. The second easiest to classify object was spots because spots are among the best-defined potential shapes that can be found in the data set. The next two classes, track and worm, were more problematic. These two classes are most often confused with each other due to the subjectivity of the judgement of specialists assigning images to those two classes. In case of the network using the VGG16 feature extractor, nearly 15.10% ± 9.01% of tracks were incorrectly assigned to the worm class, while 22.64% ± 10.70% of worms were incorrectly assigned as tracks. As shown in Figure 6, the difference between tracks and worms is very subjective: there is not much visible difference between a track (Figure 6b) and a worm (Figure 6g). It was difficult to guess the judges’ reasoning in this case. Worms were confused with artefacts: in case of VGG16, incorrect classification between those classes was 8.64% ± 4.19%. This situation was also caused by judges’ subjectivity. Due to the MSE being quite low, the proposed architecture correctly models the judges’ decision despite there only being five judges and the shape of the worm class was not clearly defined (see Section 2.3). There are two possible solutions to overcome this problem. The first is to increase the number of judges and to define each class more precisely; however, this does not guarantee improving the true positive rate of worm and track classes. The second possibility is to apply the threshold scheme (8). Application of this scheme involves a trade-off between the accuracy and the number of objects that can be classified. As shown in Table 8, Table 9 and Table 10, even the application of the lowest considered threshold t=0.50 improves the true positive rate of all classes (compare with Table 3). For example, the true positive rate of the worm classes improved from 62.59% ± 9.9% to 75.87% ± 13.51% when t=0.50, to 79.54% ± 10.97% when t=0.75, and to 89.65% ± 15.52% when t=0.90. This operation, however, results in 56.98% ± 16.92%, 34.71% ± 7.93%, and 18.445% ± 7.22% of worms being appropriately classified, respectively. Due to this finding, threshold *t* has to be chosen carefully, considering many factors of certain detection. At this moment, it is difficult to compare our results directly with those from Winter et al. [1], mainly because the DECO dataset is not publicly available; however, the accuracy of the results we obtained is very similar to those previously reported: spots 98.9% (our result from Table 10 is 98.7%), tracks 95.4% (ours: 88.9%), worms 92.9% (ours: 90.0%), and artefacts 98.2% (ours: 97.7%). Notably, we did not exclude any object either from the training or validation dataset due to labeling disagreement between judges, as was performed for the DECO dataset. Certainly, the image quality and the labelling process of the dataset have considerable impacts on the results of a method. In our case, we used approximation rather than a classification approach in DNN training, which seems to be reasonable with the presence of uncertainty in class assigning. Based on our experience, we think that unless some standardized approach to class assigning is established, uncertainties are inevitable. Therefore, the classification model should not only be able to deal with them but also take advantages of them, as does our proposed method.

## 5. Conclusions

Based on the research presented in this paper, we conclude that the proposed recognition algorithm based on the approximation of uncertain class assignment with a deep convolutional neural network together with threshold scheme seems to be promising method to identify various classes of cosmic ray images obtained from CMOS sensors used in mobile phones. We recommend using VGG16 as the feature extractor. The performance of our method using VGG16 is not considerably different from other CNN networks beside MobileNetV2. According to Table 2, both VGG16 and DenseNet201 have the smallest mean squared error; however, DenseNet201 has a more complex architecture that affects its performance. Increasing the complexity and depth of artificial neural networks for classification is not always necessary to achieve state-of-the-art results [49]. The appropriate choice of threshold *t* highly depends on the detection setup, because it is a trade-off between the accuracy and number of objects that can be classified. Because the proposed approach is based on machine learning, a high-quality training data set is a crucial component to obtain reliable classification. To improve the obtained results, a larger data set of images that contains more objects labelled by a larger number of scientists must be created. Moreover, we think that VGG16 might be a too-extensive architecture for features extraction. After acquiring the larger data set that we mentioned above, research should be conducted to optimize the CNN to reduce the number of layers and weights. A smaller CNN architecture will result in the acceleration of training and computation speed and will make the model more portable by limiting the amount of required memory to store all its parameters.

## Figures and Tables

**Table 1:** Distribution of votes for the certain class in the data set. Columns represent number of voices and rows represent certain classes. A judge could skip voting for a certain image if they were unsure as to which class it should be assigned.

	0	1	2	3	4	5
Spots	1790	103	82	34	80	261
Tracks	1832	136	91	73	109	109
Worms	1834	198	85	98	104	31
Artefacts	1115	82	3	0	0	1150

**Table 2:** Recognition rate and mean square error of networks with various input convolutional architectures.

Input Convolutional Architecture	Recognition Rate	Mean Squared Error
VGG16	85.79 ± 2.24	0.03 ± 0.00
NASNetLarge	81.66 ± 2.53	0.04 ± 0.01
MobileNetV2	78.43 ± 2.06	0.05 ± 0.01
Xception	81.49 ± 2.94	0.04 ± 0.01
DenseNet201	84.68 ± 1.77	0.03 ± 0.00

**Table 3:** Confusion matrix of the network with input convolutional VGG16 architecture.

	Spots	Tracks	Worms	Artefacts
Spots	90.84 ± 4.16	1.38 ± 1.88	5.04 ± 2.82	2.74 ± 1.78
Tracks	7.46 ± 3.80	73.31 ± 11.50	15.10 ± 9.01	4.14 ± 2.53
Worms	6.14 ± 6.01	22.64 ± 10.70	62.59 ± 9.92	8.64 ± 4.19
Artefacts	2.54 ± 1.23	0.80 ± 1.05	2.70 ± 1.60	93.97 ± 1.93

**Table 4:** Confusion matrix of the network with the input convolutional NASNetLarge architecture.

	Spots	Tracks	Worms	Artefacts
Spots	89.52 ± 5.40	3.31 ± 3.13	3.76 ± 3.29	3.41 ± 2.05
Tracks	9.27 ± 4.13	62.26 ± 5.06	17.96 ± 4.93	10.52 ± 5.46
Worms	7.83 ± 4.16	26.41 ± 9.08	51.66 ± 6.54	14.11 ± 5.30
Artefacts	2.44 ± 1.79	1.58 ± 0.39	2.71 ± 1.50	93.27 ± 2.71

**Table 5:** Confusion matrix of the network with the input convolutional MobileNetV2 architecture.

	Spots	Tracks	Worms	Artefacts
Spots	78.66 ± 3.46	13.37 ± 4.03	4.34 ± 2.70	3.63 ± 2.34
Tracks	12.28 ± 6.03	56.14 ± 5.78	24.40 ± 4.50	7.18 ± 5.96
Worms	7.15 ± 5.44	25.63 ± 9.40	50.84 ± 7.89	16.39 ± 3.87
Artefacts	2.09 ± 1.29	1.22 ± 0.61	3.15 ± 2.04	93.54 ± 1.74

**Table 6:** Confusion matrix of network with input convolutional Xception architecture.

	Spots	Tracks	Worms	Artefacts
Spots	84.29 ± 4.04	3.69 ± 2.83	6.49 ± 2.86	5.53 ± 3.67
Tracks	8.22 ± 5.66	60.21 ± 7.30	23.58 ± 7.44	7.99 ± 4.66
Worms	7.58 ± 4.77	21.18 ± 5.23	60.18 ± 8.70	11.06 ± 4.65
Artefacts	2.01 ± 1.21	1.40 ± 1.09	3.14 ± 1.71	93.45 ± 2.18

**Table 7:** Confusion matrix of the network with the input convolutional DenseNet201 architecture.

	Spots	Tracks	Worms	Artefacts
Spots	87.44 ± 4.97	4.79 ± 2.74	4.25 ± 2.61	3.52 ± 2.54
Tracks	5.11 ± 3.06	71.03 ± 5.43	20.33 ± 5.41	3.53 ± 3.49
Worms	6.03 ± 5.00	23.46 ± 7.71	61.90 ± 10.16	8.61 ± 3.15
Artefacts	2.63 ± 0.86	0.79 ± 0.87	1.93 ± 1.17	94.65 ± 1.86

**Table 8:** Confusion matrix of network with input VGG16 and threshold scheme (8) *t* = 0.50. Data remaining = 85.36 ± 1.79 (S 79.92% ± 4.37%; T 80.03 ± 3.40; W 56.98 ± 16.92; A 97.89 ± 1.41), recognition rate = 92.11 ± 1.92.

	Spots (S)	Tracks (T)	Worms (W)	Artefacts (A)
Spots	94.00 ± 3.87	2.14 ± 1.90	1.40 ± 2.13	2.46 ± 2.47
Tracks	1.99 ± 2.14	82.64 ± 5.11	14.07 ± 4.40	1.30 ± 2.38
Worms	2.65 ± 5.33	17.09 ± 10.25	75.87 ± 13.51	4.39 ± 4.15
Artefacts	1.01 ± 1.12	1.01 ± 0.69	1.86 ± 0.86	96.12 ± 1.27

**Table 9:** Confusion matrix of the network with input VGG16 and threshold schema (8) *t* = 0.75. Data remaining = 74.26 ± 2.56 (S 63.12% ± 4.63%; T 55.60 ± 5.83; W 34.71 ± 7.93; A 96.46 ± 2.08), recognition rate = 94.95 ± 1.00.

	Spots (S)	Tracks (T)	Worms (W)	Artefacts (A)
Spots	96.23 ± 2.29	0.92 ± 1.49	0.36 ± 1.13	2.49 ± 2.38
Tracks	0.83 ± 2.64	88.96 ± 6.79	8.72 ± 5.25	1.49 ± 3.46
Worms	1.00 ± 3.16	14.24 ± 11.74	79.54 ± 10.97	5.22 ± 5.75
Artefacts	0.65 ± 0.64	0.56 ± 0.48	1.80 ± 0.81	97.00 ± 0.95

**Table 10:** Confusion matrix of the network with input VGG16 and threshold schema (8) *t* = 0.90. Data remaining = 62.60 ± 2.88 (S 42.03 ± 5.65; T 29.91 ± 6.55; W 18.445 ± 7.22; A 94.97 ± 2.17), recognition rate = 96.95 ± 1.38.

	Spots (S)	Tracks (T)	Worms (W)	Artefacts (A)
Spots	98.71 ± 2.99	0.45 ± 1.44	0.00 ± 0.00	0.84 ± 1.78
Tracks	1.43 ± 4.52	88.89 ± 12.78	8.85 ± 8.78	0.83 ± 2.64
Worms	0.00 ± 0.00	7.43 ± 15.82	89.65 ± 15.52	2.92 ± 6.23
Artefacts	.38 ± 0.50	0.27 ± 0.44	1.64 ± 0.81	97.70 ± 1.24

## Data Availability

Both source codes and data are available for download from the online repository https://github.com/browarsoftware/credo_dnn_rays_recognition, accessed on 10 March 2021.
